# The effect of mobile applications for improving adherence in cardiac rehabilitation: a systematic review and meta-analysis

**DOI:** 10.1186/s12872-019-1149-5

**Published:** 2019-07-12

**Authors:** Linqi Xu, Feng Li, Changli Zhou, Jinwei Li, Chengcheng Hong, Qian Tong

**Affiliations:** 1 School of Nursing, Jilin University, Changchun, 130000 China; 2 School of Health Sciences University of Manchester, M13 9PL, Manchester, UK; 3 The First Hospital of Jilin University, No 71, Xin Min Avenue, Changchun, 130000 China

**Keywords:** Cardiac rehabilitation (CR), Mobile application, Adherence

## Abstract

**Background:**

Despite of the established effectiveness, the acceptance and adherence of cardiac rehabilitation (CR) remains sub-optimal. Mobile technologies are increasingly used in promoting CR without any firm evidence of their safety and efficacy. This systematic review and meta-analysis were aimed to assess the effect of mobile applications as an intervention for improving adherence to CR.

**Methods:**

Relevant studies were searched in PubMed, the Cochrane Library, Embase and Web of Science from inception to 29th December 2018. Eligible studies were the ones which used mobile applications as a stand-alone intervention or as the primary component for the intervention directed at improving CR adherence, without any limitations on outpatient or home-based CR.

**Results:**

Eight studies were eligible for the systematic review including four randomized controlled trials (RCTs) as well as four before-after studies of which only one had control group. Four RCTs and 185 patients in experimental group were included in meta-analysis, which had evaluated the effect of mobile health applications on CR completion and had reported that the adherence of patients using mobile applications was 1.4 times higher than the control group (RR = 1.38; CI 1.16 to 1.65; *P* = 0.0003). Moreover, we also found mixed results in exercise capacity, mental health and quality of life.

**Conclusion:**

The use of mobile applications for improving the adherence of the CR might be effective. However, it appears to be in the initial stage of implementing mobile applications in CR and more research is essential to validate their effectiveness.

## Background

Cardiovascular disease (CVD) is the leading cause of mortality worldwide, with an increase of 14.5% from 2006 to 2016 [1]. As CVD is the major burden of non-communicable disease, decreasing CVD-induced morbidity and mortality has been recognized as a key global health priority for the World Health Organization (WHO) [2]. According to the American Heart Association (AHA)/ American College of Cardiology (ACC) [3, 4] and the European Society of Cardiology (ESC) [5], cardiac rehabilitation (CR) is a class IA recommendation for patients with CVD and has been demonstrated to reduce cardiac and all-cause mortality, fewer cardiac events and less re-hospitalization [6–9]. Despite of the proven benefits and guideline recommendations, the uptake and adherence remains sub-optimal in almost all countries wherever CR is available, with only 30% of eligible patients participating in the UK and the USA [10–12], approximately 30% of eligible patients in Canada, and a little higher at around 50% in the rest of European countries [13]. The obstacles to participation include different factors such as limited program availability, transport restrictions, inconvenient program scheduling, and cost [14]. Lately, the ACC and AHA has emphasized on updating the existing CR measure in order to improve CR participation [15]. It is clear that the traditional CR does not meet the needs of many eligible patients, and hence improvement in CR program is required to enhance its utilization.

Mobile technology has the potential to overcome barriers to deliver CR and may be a useful tool for promoting adherence. Mobile health applications are increasingly recognized for multiple benefits as mentioned below: (1) To receive health related knowledge and automated feedback information, (2) To review sports records, and (3) To interact with other users or healthcare providers, which may be helpful to promote physical activity. Studies have shown that use of mobile health (mhealth) interventions have positive benefits in increasing motivation and participation in rehabilitation [16]. Recent meta-analysis have reported the efficacy of mhealth technologies on CR. However, the interventions of most of the studies comprised fixed-line telephone, biosensors and short message service (SMS). The interventions also included websites which represented an older model of CR delivery without any interactive feedback, and did not find any significantly inferior outcomes in CR adherence [17, 18]. Other systematic review analyzed mobile applications, which were aimed at prevention of CVD, and reported that mobile applications have the potential to improve access to CR [19, 20]. Therefore, it is unclear if mobile applications provide any benefit in improving referral, adherence and functional capacity to CR. In order to address this, we performed a systematic review and meta-analysis aimed to compare the effectiveness of mobile application-based CR with traditional CR, in terms of the adherence, exercise capacity and other related outcomes.

## Methods

### Literature search

We searched for candidate studies in PubMed, the Cochrane Library, Embase and Web of Science from inception to 29 December 2018. The following terms were searched: mobile applications, mobile app, internet app, web app, portable electronic application, portable electronic app, portable software application, smart phone, mobile health, and CR. Additionally, other references from published literatures which met the inclusion criteria were also identified by searching relevant systematic reviews and meta-analyses manually CR. There were not any restrictions regarding language or date while searching the articles. However, studies which were only published as an abstract or without any results were not included.

### Inclusion/exclusion criteria

In order to be included in the current meta-analysis, studies had to meet the following PICOS criteria.

1. Patients: The patients with CVD and the patients who were eligible for CR were included in this study with no restrictions on outpatient or home-based CR.
2. Intervention: The studies which used mobile applications as a stand-alone intervention or as the primary component for the intervention directed towards improving CR adherence were included. If the studies used only SMS or used only a web site or attend a virtual cardiac rehabilitation program (v-CRP), or used only video conferencing or telephone calls between patient and health provider alone, they were excluded.
3. Comparisons: The controlled group received traditional rehabilitation without mobile applications or wearable devices.
4. Outcomes: The primary and secondary outcomes were observed. The primary outcome of interest was adherence of CR. And the secondary outcomes were exercise capacity, mental health and quality of life.
5. Study design: Both randomized as well as non-randomized studies were eligible for inclusion. There were not any restrictions on sample size or follow-up duration. However, the qualitative studies were excluded.

### Study selection

Once the titles and/or abstracts of articles were identified through the above mentioned search strategy, two reviewers screened the identified articles independently to determine whether the selected articles could potentially meet the inclusion criteria. Once the studies were selected, two reviewers obtained and evaluated the relevant full-text articles to confirm if the articles met the inclusion criteria. If there were any concerns or controversies about the eligible selected studies, a third reviewer stepped in to resolve these issues and helped to reach to a final agreement.

### Data exaction and study quality

Study characteristics were obtained independently by two authors with the help of a standardized electronic data collection form. The collected data provided following information: names of the first authors, publication year, characteristics of the participants, sample size, study setting, follow-up durations and description of the interventions.

As per the tool for risk of bias mentioned in the Cochrane Handbook for Systematic Reviews of Intervention, the quality of four randomized controlled studies were evaluated by two authors. The tool for risk of bias included selection, performance, detection, attrition, reporting, and other biases. The risk of each bias was classified as “unclear,” “low,” or “high”. The four quasi-experimental studies were evaluated as per the criteria published by the Australian Evidence-Based Health Care Center (2016) [21]. There were nine items for the remaining quasi-experimental studies. Each criterion was evaluated and marked as “yes”, “no”, “unclear”, or “not applicable”. At the end, the overall quality of each articles were rated by each reviewer as A (high quality), B (medium quality), or C (low quality).

### Data analysis and synthesis

A total of eight studies were evaluated in the systematic review. A total of four studies were included for the meta-analysis. And this included the number of participants who completed CR from both traditional CR group and mobile application-based CR group with a fixed-effects model. Additionally, other studies which had reported similar outcomes were also used for the analysis. We used risk ratio (RR) as the primary summary measure and mean difference (MD) as the second summary measure. Meta-analysis was not conducted if the study did not exhibit standardized mean difference (SMD) or standard error of the mean (SEM). The RRs and MDs were both calculated with 95% confidence intervals (CIs), and statistical significance was achieved with a *P* value of < 0.05. Further, we also tested for heterogeneity using the *I*^*2*^ test whose values greater than 50% indicate a high heterogeneity for the latter. Review Manager 5.2 was used to perform all statistical analyses.

## Results

### Search results

**Fig. 1:**
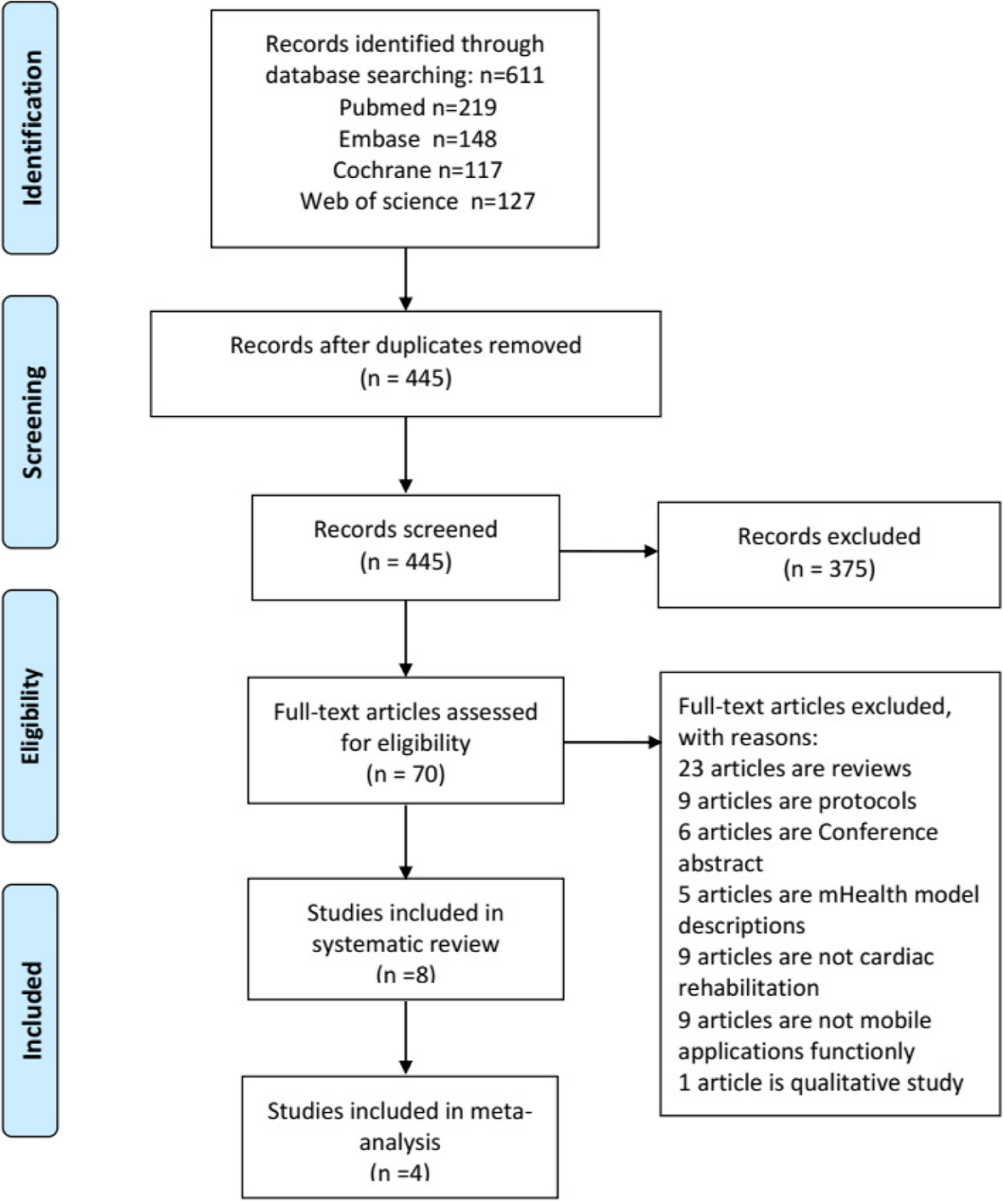
Flow Diagram of Search Results

As per our search criteria, we first identified a total of 611 potential articles. However, after removing the duplicate articles, we had 445 articles. Further, after screening the titles and/or abstracts, additional 375 manuscripts were excluded from the above mentioned 445 articles. Next, the full-text articles of remaining 70 studies were reviewed and evaluated. After careful considerations, 62 out of the 70 articles were excluded due to following reasons: 23 articles were review articles, 9 articles were protocols, 6 articles were conference abstracts, 5 articles were mHealth model descriptions, 9 articles were not on CR, 9 articles did not include any mobile applications intervention, and 1 article was a qualitative study. As a result, at the end, a total of 8 studies/articles were eligible and were included in our systematic review and 4 randomized controlled trials (RCTs) were included for the meta-analysis (Fig. 1).

### Study characteristics

A total of eight studies from four different countries were included in this study as mentioned in Table 1. A total of four studies were evaluated in meta-analysis. The selected studies were from three different types: four randomized controlled trials/studies [25–27, 29], three non-controlled before-after studies [22–24] and one controlled before-after study [28]. When all studies were considered together, a total of 506 participants were evaluated. Out of these 506 participants, 18% of the participants were female. The age of participants ranged from 25 to 73 years old. Participants with different types of CVD were absolutely eligible for CR.

**Table 1:** Study Characteristics

Author and Country	Study design	CVD population	Sample size	Mean age	%female	Intervention/Control	Intervention duration
Forman et al.2014, USA [22]	quasi-experimental study	phaseIICR	26	59 (43–76)	23	I: HC mobile app + CR no controlled	30 days
Harzand et al.2018, USA [23]	quasi-experimental study	CHD referred to CR	21	65	0	I: commercially available smartphone platform+ CR; no controlled	12 weeks
Laustsen et al. 2018, Denmark [24]	quasi-experimental study	CR	34	58 (25–72)	18	I: SportsMedicin app with HR monitoring + CR; no controlled	12 weeks
Rosario et al.2018, Australia [25]	RCT	CR	66	ns	ns	I: STAHR app with health monitoring + CR; C: TCR	6 weeks
Skobel et al.2017, German [26]	RCT	phaseIICR	118	59 (45–73)	11	I: GEx system intervention + CR; C: TCR	6 weeks
Varnfield et al.2014, Australia [27]	RCT	post-MI referred to CR	94	55	13	I: CAP-CR; C: TCR	6 weeks
Widmer et al.2015, USA [28]	Controlled, non-Randomized before–after study	ACS referred to CR	76	66	27	I_1_: PHA mobile app + CR; I_2_: PHA mobile app + P-CR; C_1_: TCR; C_2_: P-TCR	3 months
Widmer et al. 2017, USA [29]	RCT	ACS referred to CR	71	63	18	I: PHA mobile app + TCR; C: TCR	3 months

Key: *CVD* Cardiovascular Disease, *CR* Cardiac Rehabilitation, *I* Intervention Group, *C* Controlled Group, *RCT* Randomized Controlled Trial, *CHD* Coronary Heart Disease, *MI* Myocardial infarction, *ACS* Acute Coronary Syndrome, *NS* Not Specified, *HC* Heart Coach, *STARHR* Smartphone Technology and Heart Rehabilitation, *GEx* Guide Exercise, *APP* Application*, CAP* Care Assessment Platform, *PHA* Personal Health Assistant, *TCR* Traditional Cardiac Rehabilitation, *P-CR* Post Cardiac Rehabilitation, *HR* Heart Rate

As mentioned in Table 2, the included articles/studies basically described the applications as mix of mobile interventions [22–27] or a stand-alone intervention [28, 29]. Different studies had used different types of interventions. For example, four studies [22, 23, 26, 27] included web dashboards and four studies [24–27] used wearable devices. Moreover, in order to support the patients while recording and monitoring their own physical status as well as to provide feedback, a total of seven applications were installed on different smartphones with either android or iOS operating systems.

**Table 2:** Intervention Characteristics

Author and Country	Key components of intervention	Function of mobile applications	Application terminal
Forman et al.2014, USA [22]	HC app; HC-based Web dashboard	a to-do list with medications, walking, education, and surveys; tracking of physical activity; feedback from clinician	iPhone (version 3 or higher), an iPad, or an iPod touch (version 4 or higher)
Harzand et al.2018, USA [23]	smartphone platform smartphone app; hospital --facing online dashboard	remote patient monitoring; care coordination by a trained cardiology PA	Samsung Galaxy S4 or comparable
Laustsen et al. 2018, Denmark [24]	SportsMedicin app; HR monitor (Zephyr BioHarnessTM)	remote patient monitoring training intensity	Sony Xperia
Rosario et al.2018, Australia [25]	STAHR app; BP monitor and digital weight scale	receive feedback with activity through the app	Samsung Galaxy SIII (SG3)
Skobel et al.2017, German [26]	GEx system: smartphone app wearable sensor measuring information of one-lead ECG, HR, respiration rate and activity level; web-based tool for medical professionals	exercise guiding; feedback from clinician remote patient monitoring; be alerted in case of problems	No specified
Varnfield et al.2014, Australia [27]	CAP-CR: StepCounter app; health monitor of step counter, BP weight; web for clinician	motivational and educational materials delivering; remote patient monitoring; feedback from clinician	No specified
Widmer et al.2015, USA [28]	PHA app	daily tasks for healthy lifestyle behaviors; tracking of	No specified
Widmer et al.2017, USA [29]		progress, log weight, BP, lab values, daily activity, diet; feedback from clinician	

Key: *HC* Heart Coach, *APP* Application, *PA* Physician Assistant, *STAHR* Smartphone Technology and Heart Rehabilitation, *GEx* Guide Exercise, *HR* Heart Rate, *ECG* Electrocardiogram, *CAP* Care Assessment Platform, *BP* Blood Pressure, *PHA* Personal Health Assistant

### Assessment of study quality

**Fig. 2:**
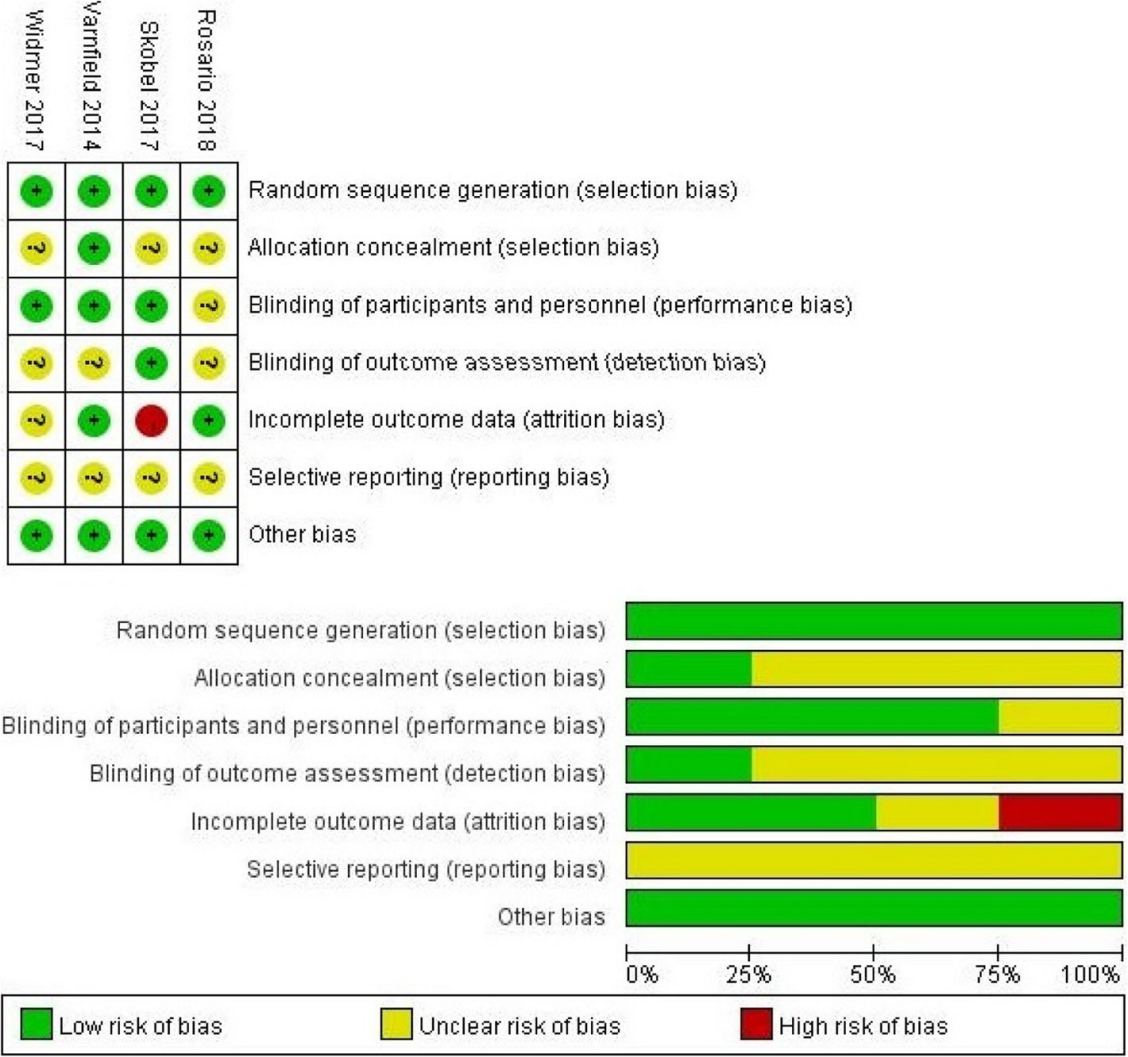
Risk of Bias Assessment

As mentioned in Fig. 2, the quality of four RCTs [25–27, 29] were summarized by using the Cochrane’s risk of bias table of Review Manager 5.2. Further, a total of four studies [25–27, 29] correctly reported the generation of random sequences and only one study [27] explicitly mentioned that the allocation was concealed. Participants were blinded in three of the selected studies [26, 27, 29]. However, one study [25] could not be conducted with perfect blinding. Moreover, three studies [25, 27, 29] did not report whether the outcomes were affected due to the blindness of the participants and only one study [26] reported that the outcome was not affected at all by the lack of blindness. It was also documented that the integrity of the outcomes was low risk in two studies [25, 27], unclear in one study [29] and high risk in one study [26]. Additionally, for reporting bias, four studies [25–27, 29] were assessed as unclear on the presence of bias.

Table 3 describes the quality of four quasi-experimental studies [22–24, 28]. Three [22–24] out of the four quasi-experimental studies did not have any control group and only one study [28] had included a control group. Moreover, these articles essentially met the other evaluation criteria. Regarding the quality of the articles, two articles [22, 28] received an A (high quality) rating and two articles [23, 24] received a B (medium quality) rating.

**Table 3:** Quality evaluation results for quasi-experimental studies

Study	1	2	3	4	5	6	7	8	9	Comments
Forman DE	Yes	Yes	Yes	No	Yes	Yes	Yes	Yes	Yes	A
Harzand A	Yes	Yes	Yes	No	Yes	Yes	Yes	Yes	Yes	B
Laustsen L	Yes	Yes	Yes	No	Yes	Yes	Yes	Yes	Yes	B
Widmer RJ	Yes	Yes	Yes	Yes	Yes	Yes	Yes	Yes	Yes	A

Key: 1: Clear description of “cause” and “effect”; 2: Similar participants included in comparisons; 3: Similar treatment or care other than the exposure in comparisons; 4: There was a control group; 5: Multiple measurements of the outcome; 6: Complete follow-up or strategies to deal with incomplete follow up; 7: Outcome measured in the same way for comparisons; 8: Outcome measured in a reliable way; 9: Appropriate statistical analysis

### Effect on CR adherence

**Fig. 3:**
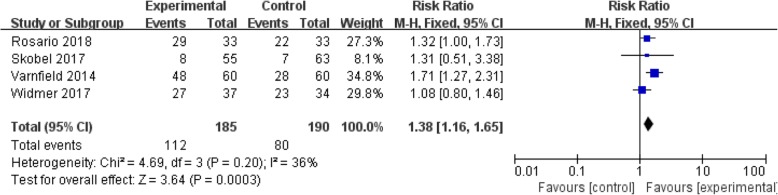
CR Completion for Intervention Group Compared with Control Group

A total of eight studies (Table 4) evaluated the effect of mobile health applications on CR adherence and four RCTs were included in meta-analysis. As mentioned in Fig. 3, meta-analyses and RR calculations have indicated the CR completion and CR adherence. These results indicated that compare to traditional CR, the CR completion was 1.38 times higher in mobile application based CR (RR = 1.38; CI 1.16 to 1.65; *P* = 0.0003).

**Table 4:** Summary of selected outcomes by study design

Author				Quasi-experimental studies			
	Numbers of studies that assessed this	Randomized controlled trial		Controlled before-after study		Uncontrolled before-after study	
Outcome	Outcome	Study	Effect	Study	Effect	Study	Effect
CR adherence	7	Rosario et al. 2018 [25]	+++	Widmer et al. 2015 [28]	–	Forman et al. 2014 [22]	∫
		Skobel et al. 2017 [26]	+			Harzand et al. 2018 [23]	∫
		Varnfield et al. 2014 [27]	+++			Laustsen et al. 2018 [24]	∫
		Widmer et al. 2017 [29]	++				
Exercise capacity	7	Rosario et al. 2018 [25]	^	Widmer et al. 2015 [28]	+++	Harzand et al. 2018 [23]	+++
		Skobel et al. 2017 [26]	+++			Laustsen et al. 2018 [24]	+++
		Varnfield et al. 2014 [27]	+				
		Widmer et al. 2017 [29]	^				
Mental health	5	Rosario et al. 2018 [25]	–	Widmer et al. 2015 [28]	+++		
		Skobel et al. 2017 [26]	–				
		Varnfield et al. 2014 [27]	+++				
		Widmer et al. 2017 [29]	++				
QoL	3	Skobel et al. 2017 [26]	–	Widmer et al. 2015 [28]	+++	Laustsen et al. 2018 [24]	+++
		Varnfield et al. 2014 [27]	+++				
		Widmer et al. 2017 [29]	+++				

Key: *QoL*: Quality of Life; *CR*: Cardiac Rehabilitation

+++: statistically significant effect; ++: greater improvement in intervention group than control but between group difference not significant; +: significant improvement in both groups but between group difference not reported or not significant; −: no reported change between treatment groups; ^: within-group improvement not significant; ∫: adherence improvement data from participant survey

Four quasi-experimental [22–24] studies evaluated the CR adherence with mixed results. The controlled before-after study [28] reported that there was not any difference between the intervention and controlled groups for the number of CR sessions (29.0 + 3.8 vs. 30.7 + 4.1, *P* = 0.77). Three studies [22–24] indicated that the mobile applications had positive impact on CR compliance and adherence.

### Effect on exercise capacity

**Fig. 4:**
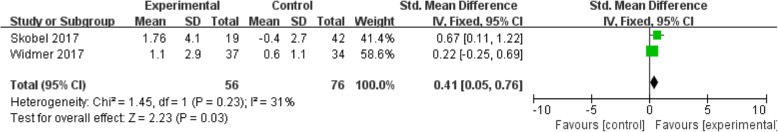
Forest plot for the change of VO2peak

Changes in exercise capacity were also evaluated in four RCTs and three quasi-experimental studies. All these studies have reported mixed results. The four RCTs used different types of exercise capacity measurements. Two studies [26, 29] used peak oxygen uptake (VO_2peak_) and two studies [25, 27] used 6-min walk test (6MWT). We did not perform meta-analyses and forest plot for 6MWT [25, 27] due to insufficient data, so meta-analyses was conducted only for VO_2peak_ [26, 29]. As presented in Fig. 4, the results indicated that VO_2peak_ was improved in intervention group compared with control group (SMD = 0.41 ml/min/kg; CI = 0.05–0.76; *P* = 0.03). One study [27] reported that both study groups improved the 6MWT from baseline to week 6 and then maintained this improvement to month 6. However, there was not any statistically significant difference between both groups. In addition, another study [25] reported that there was not any significant difference in exercise capacity between two groups.

A controlled before-after study [28] used the Bruce Protocol treadmill test and found that the intervention group had a significant improvement in in exercise capacity (2.5 + 2.7 ml O2/min per kg, *p* = 0.004). Moreover, in one quasi-experimental study, a significant improvement of 1.0 metabolic equivalents in mean functional capacity was also reported [23]. Additionally, Laustsen et al. had reported that the VO_2peak_ was significantly increased by 10% after 12-week tele monitored exercise-based CR [24].

### Effect on mental health and quality of life

Five studies assessed mental health of the participants by different methods (DASS21 depression, anxiety and stress [25, 27]; HADS-anxiety, depression [26]; exercise stress testing [28, 29]) and reported mixed results. Two RCTs [27, 29] and one controlled before-after study [28] evaluated psychosocial wellbeing and reported that there were improvements in patients using mobile apps. However, there was not any statistically significant difference in anxiety and depression between intervention and control groups in another two RCTs [25, 26].

Quality of Life (QoL) was assessed in five studies using three different instruments (EuroQol-5-Dimensions (EQ-5D) [26, 27], Dartmouth QoL survey [28, 29], 36-Item Short Form Health Survey (SF-36) [24]). Mixed and unclear results were reported. Two RCTs [27, 29] and one controlled before-after study [28] evaluated the mobile application based CR participants and reported that they showed significant improvement in QoL compared with traditional CR. Laustsen et al. [24] also found that there was a significant improvement in physical and mental health-related quality of life (HRQoL) in the intervention group. However, there was not any significant difference in EQ-5D between intervention and control group in one of the RCTs [26].

## Discussion

Our results have indicated that mobile applications in CR may improve the adherence of CR in current scenario. The adherence of CR may affect the patients’ physical and psychological conditions. Therefore, we also analyzed the effect on exercise capacity, mental health and QoL. However, our findings have also revealed mixed results regarding the effectiveness across outcomes in exercise capacity, mental health and QoL. The evidence was modest; however, it was limited due to very small number of studies, diverse populations, different length of CR and other methodological considerations, mainly for the statistical analysis.

It was also reported that the usage of mobile applications may have a statistically significant improvement on adherence of CR. This result was parallel with another review which had evaluated the efficiency of telehealth CR delivering by using a mobile phone, SMS and website [16]. Compared with these traditional old model of CR, mobile applications are considered simple and easy to perform as well as satisfactory intervention, which is much more flexible to deliver exercise training. Hence, we evaluated the attractive functions that might be useful in promoting CR within this review. First of all, intervention designs with up-to-date theoretical base are generally more effective than those without any substantial and important theoretical framework. Moreover, based on a behavior change framework, the Fogg’s Persuasive System Design principles, the mobile applications can be designed such that they can promote education and help regarding self-management in patients to motivate them for CR. Most of the mobile applications [22, 26–29] which were included in our systematic review could support rehabilitation through education and promoting better exercise habits. And this has been proved to transform beliefs associated to risk behavior into ideal behaviors to improve physical activity and exercise [30]. Second, mobile applications in our study that could set sports goals and also provide personalized guidance with feedback were demonstrated to be more beneficial in promoting physical activity [31]. Most of the participants also reported positive experience towards the feedback which could lead to promotion of social support as well as increase the motivation. Third, mobile applications provide opportunities to share the data with the health care providers which has the potential to promote CR through subjective norms and social influence [32]. It has been reported that many patients had expected to share the obtained data with their health care providers and they viewed this as a key benefit of using mobile applications [33]. Another study has reported great promotion of participation in CR through interaction, communication and feedback [34]. Additionally, communication may also be beneficial in improving psychosocial well-being and quality of life of the patients. Further, the mobile applications are generally designed on the basis of the behavior change framework which includes automated feedback information, sport record reviewing and interaction with other users or healthcare providers. And this might be an important as well as beneficial tool to improve the adherence of CR.

Mobile applications will continue to advance and are anticipated to be robust ubiquitous devices in the future. Researchers should also think about how mobile applications can be effectively used in future to promote the implementation of CR. Moreover, gaining a deeper understanding of user experience would also help to optimize the relevance as well as the utility of the mobile applications. This review has evaluated following various barriers on why participants discarded the mobile applications: (1) Was it because the wearable sensor was not very comfortable? (2) Was it due to safety algorithms or technical errors? (3) Was it difficult to use? Most of the research in our study focused on the functions and applications of mobile applications. There were only few studies which focused on security issues from patients. Studies have reported that a sense of distrust regarding the security of data can also affect a patient’s enthusiasm for using an application [19]. Thus, future research should also focus on the security aspects of mobile application data in order to fully protect patient’s privacy. As patients with CVD are generally elderly, they may prefer limited use of mobile phones. Thus, in order to motivate them, it is also important to develop a reliable, low-cost, and user-friendly mobile application.

Our current study has several limitations. First, the sample size from all the four RCTs is very small. There are less than 400 patients that were included in meta-analyses and less than 200 patients in experimental group which may cause a bias. Hence, the evidence may not be sufficiently strong to determine if mobile applications are effective to improve adherence of CR. Second, even though we aimed to get the completed outcomes, the evidence was too trivial and limited to demonstrate the outcomes of exercise capacity, mental health, and QoL, predominantly due to insufficient data in the original articles. Furthermore, we did not perform meta-analysis and forest plot for mental health and QoL because multiple distinctions between instruments and the evaluation of different health dimensions made the mental health and QoL difficult to measure, and caused high heterogeneity. Third, most of the RCTs compared an intervention directly with the “traditional cardiac rehabilitation (TCR)” whose details were not described. Fourth, differences in types of mobile applications, sample size, and follow-up duration among included studies might be a major cause of the heterogeneity. Fifth, in the seven of the studies in this review the mean age of the participants was 60 years old, which may be due to a bias in selection process. Sixth, most of the included studies in this review were conducted using medical registry databases, which suffer from an intrinsic risk of coding imprecisions and/or incompleteness. Seventh, the relative risk values from the included studies were basically adjusted to control for a wide range of confounding factors such as demographics, lifestyle, and clinical factors, and this may possibly influence experimental outcomes.

## Conclusion

Our findings have demonstrated that there might be a significant increase in adherence of CR among patients with CVD. Mobile applications have a great potential to improve CR delivery and minimize health problems. However, the implementation of the mobile applications in CR seems to be in the initial stage. There is a need of more intelligent wearable devices which has to be explored and combined with mobile applications to form a closed CR management system in the future. Additional research is also required to verify their effectiveness.

## Data Availability

Studies included in this systematic review are available online.

## Abbreviations

6MWT: 6-min walk test
ACC: American college of cardiology
AHA: American Heart Association
CI: Confidence interval
CR: Cardiac rehabilitation
CVD: Cardiovascular disease
EQ-5D: EuroQol-5-dimensions
ESC: European society of cardiology
HRQoL: Health-related quality of life
MD: Mean difference
mHealth: Mobile health
QoL: Quality of life
RCT: Randomized controlled trial
RR: Risk ratio
SEM: Standard error of the mean
SF-36: 36-item short form health survey
SMD: Standardized mean difference
SMS: Short message service
TCR: Traditional cardiac rehabilitation
v-CRP: Virtual cardiac rehabilitation program
VO_2peak_: Peak oxygen uptake
WHO: World Health Organization
